# The impact of multiple climatic and geographic factors on the chemical defences of Asian toads (*Bufo gargarizans* Cantor)

**DOI:** 10.1038/s41598-019-52641-4

**Published:** 2019-11-21

**Authors:** Yueting Cao, Keke Cui, Hongye Pan, Jiheng Wu, Longhu Wang

**Affiliations:** College of Pharmaceutical Sciences, Zhejiang University, Hangzhou, 310058 China

**Keywords:** Evolutionary ecology, Animal physiology

## Abstract

Chemical defences are widespread in nature, yet we know little about whether and how climatic and geographic factors affect their evolution. In this study, we investigated the natural variation in the concentration and composition of the main bufogenin toxin in adult Asian toads (*Bufo gargarizans* Cantor) captured in twenty-two regions. Moreover, we explored the relative importance of eight climatic factors (average temperature, maximum temperature, minimum temperature, average relative humidity, 20–20 time precipitation, maximum continuous precipitation, maximum ground temperature, and minimum ground temperature) in regulating toxin production. We found that compared to toads captured from central and southwestern China, toads from eastern China secreted higher concentrations of cinobufagin (CBG) and resibufogenin (RBG) but lower concentrations of telocinobufagin (TBG) and cinobufotalin (CFL). All 8 climatic variables had significant effects on bufogenin production (*r*_*i*_>0.5), while the plastic response of bufogenin toxin to various climate factors was highly variable. The most important climatic driver of total bufogenin production was precipitation: the bufogenin concentration increased with increasing precipitation. This study indicated that the evolution of phenotypic plasticity in chemical defences may depend at least partly on the geographic variation of defensive toxins and their climatic context.

## Introduction

Chemical defences is ubiquitous in nature and can serve for deterring predators, competitors, parasites, and pathogens^1–3^. Many species can secrete a large diversity of toxic defensive compounds for self-protection. Amphibians are one of the most endangered species on the planet, so much attention is being paid to their chemical defences variation^4^. Some studies have shown that many amphibians can phenotypically adjust their defensive chemicals in terms of environmental fluctuations^5–7^. For example, many toads species can upregulate the synthesis of toxic chemicals to increase their unpalatability and toxicity to predators, thereby reducing their predation risk, when they receive predator cues^8,9^.

Previous research has recorded the relationship between the chemical defences of certain amphibians and the age, body size, and sex of individuals^5,10^. Many studies have also investigated predator-induced and competitor-induced plasticity in chemical defences^8,11,12^. However, no previous study has addressed whether and how climate-induced plasticity in the chemical defence of amphibians varies under different climate contexts. In addition, some studies have confirmed variations in chemical defences under different geographical conditions^13–15^, but a few research have focused on the regular pattern of chemical defence plasticity with respect to geographic variation. Therefore, a more systematic investigation of the variation in climate-induced and geography-induced chemical defences, and a comprehensive analysis of such responses will contribute to a better understanding of the ecology and evolution of phenotypic plasticity.

**Figure 1:**
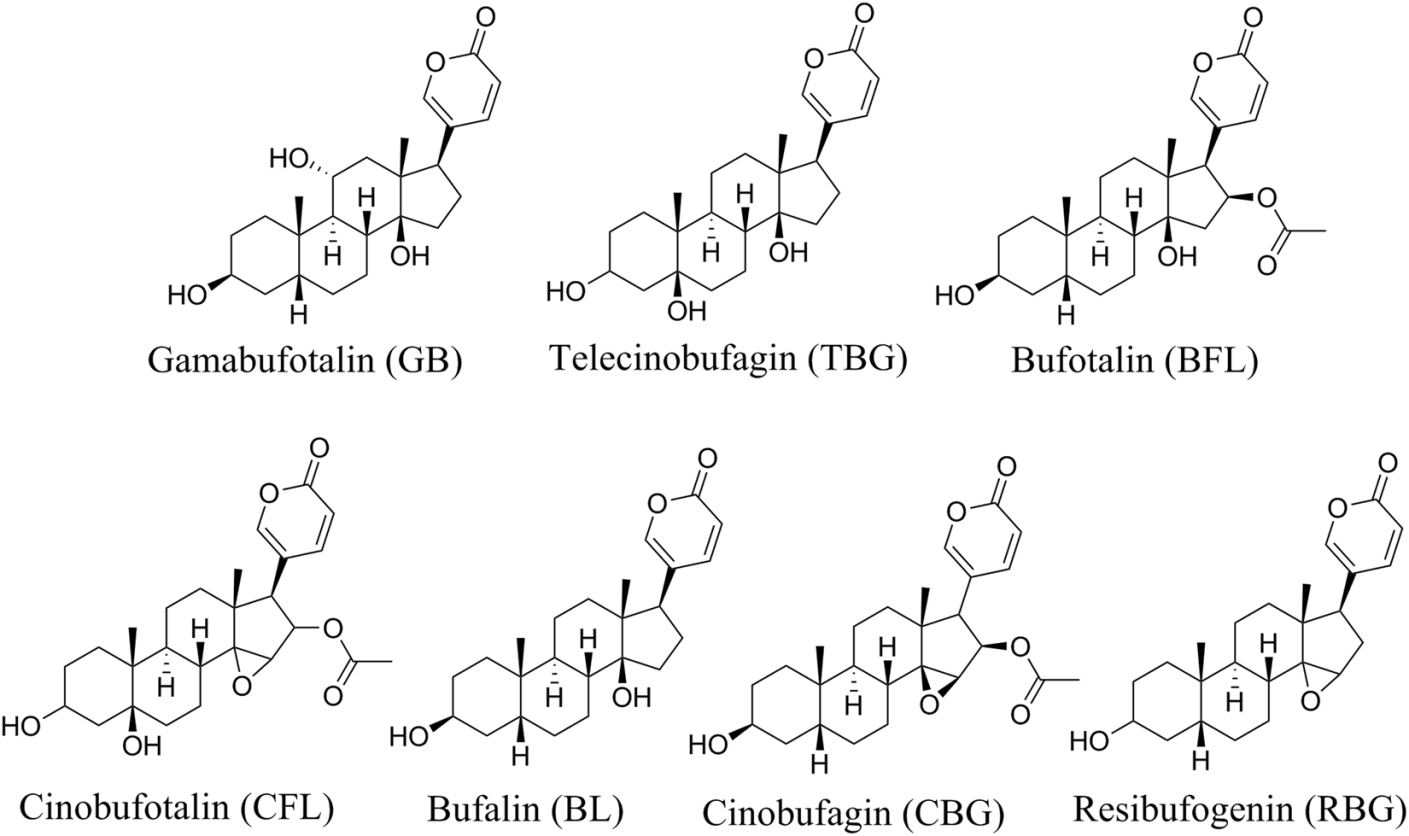
Chemical structures of seven bufogenins.

Asian toads (*Bufo gargarizans*), one of the most common anuran species in China, are widely distributed in eastern, southwestern, and central China and other regions. Similar to other bufonids, Asian toads can secrete and release highly antimicrobial and poisonous defensive toxins from large parotoid glands when they face an environmental risk^16^. These toxins are important defences against predators and microorganisms^17–19^. Many reports have confirmed that the main component of these toxin cocktails is bufadienolide, cardiotoxic steroids that inhibit Na^+^/K^+^-ATPases^20,21^. Bufadienolide compounds are usually classified into two types: free-type bufogenins and conjugated-type bufotoxins^22^. Generally, bufagenins are more toxic than bufotoxins^15,23^. More than 50 different bufagenin compounds have been identified in Asian toads to date^24,25^. Among them, gamabufotalin (GB), telocinobufagin (TBG), bufotalin (BFL), cinobufotalin (CFL), bufalin (BL), cinobufagin (CBG) and resibufogenin (RBG) (Fig. 1) are the dominant components, representing more than 80% of the total amount of bufagenins^26^. These compounds are considered major contributors to the role of toxins in defence^27^. Despite similar chemical structures, the different bufagenins may exhibit functional differences in the potency and selectivity of their toxicity to different types of predators^20,28^. For example, many arthropod Na^+^/K^+^-ATPases lack the binding site for certain bufagenins, whereas certain vertebrate Na^+^/K^+^-ATPases have high affinity for the same bufagenins^29,30^. Therefore, the concentrations and composition of such highly toxic components in the toxin blend are more relevant to the deterrent efficiency of the chemical defence than the amount of toxin^20^.

In this study, we aimed to explore the natural variation in chemical defences and its relationships with assorted climatic and geographic factors in adult Asian toads. We described the variation in chemical defences with three variables: the concentrations of single bufogenins, the total bufogenin concentration (the sum of the concentrations of the main seven bufogenins) and the constituent ratio of bufogenin compounds (relative proportions of various bufogenins). In addition, we investigated the extent of variation in these defensive toxins from different geographical regions and explored the relative importance of the influence of various climatic factors on toxin variation. Our study confirms that climatic factors may substantially regulate bufogenin plasticity and affect the particular geospatial distribution of these defensive chemicals. To our knowledge, this study is the first to determine the variation in chemical defences among Asian toads in different geographical regions and its relationship to climatic factors.

## Results

### Constituent ratios and correlation of the bufogenins

**Figure 2:**
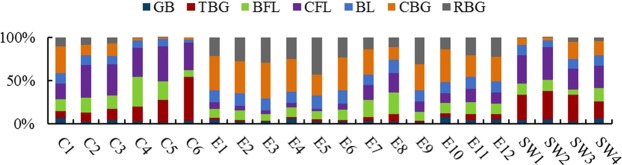
Percentage stacked column chart of seven bufogenins in different geographical origins.

The total bufogenin concentrations at the twenty-two test sites ranged from 8.43% to 19.34% (Table 1). A percentage stacked column chart of the various component concentrations is shown in Fig. 2. Clearly, the constituent ratios of the seven bufogenins from the twenty-two sites were significantly different. The highest constituent ratios of GB, TBG, BFL, CFL, BL and CBG were more than 4.0, 56.8, 5.6, 13.7, 5.4 and 31.6 times the lowest ones, respectively. However, the RBG concentration was too low to detect in Zhangjiajie, Hunan Province (Table 1).

**Table 1:** The concentration of bufogenins in Asian toads from twenty-two geographical origins.

No.	Concentration of bufogenins (%)						
	GB	TBG	BFL	CFL	BL	CBG	RBG
C1	1.12 ± 0.11^a^	1.35 ± 0.04 ^g^	2.20 ± 0.08 ^cd^	2.95 ± 0.14 ^h^	2.11 ± 0.12^de^	5.02 ± 0.12 ^g^	1.79 ± 0.03^i^
C2	0.25 ± 0.05^j^	1.24 ± 0.05 ^h^	1.97 ± 0.03^fg^	4.40 ± 0.04^b^	1.26 ± 0.03^k^	1.33 ± 0.03 ^m^	1.06 ± 0.06^k^
C3	0.45 ± 0.03^gh^	1.32 ± 0.01^gh^	1.60 ± 0.03^i^	3.78 ± 0.03^e^	1.02 ± 0.02 ^l^	1.49 ± 0.04 ^l^	0.76 ± 0.04 ^m^
C4	0.26 ± 0.03^j^	1.46 ± 0.01 ^f^	2.93 ± 0.07^b^	2.87 ± 0.07 ^h^	0.77 ± 0.04 ^m^	0.19 ± 0.03^p^	0.13 ± 0.02^n^°
C5	0.22 ± 0.05^j^	2.58 ± 0.08^e^	2.26 ± 0.05^c^	4.14 ± 0.01^c^	0.89 ± 0.09 ^lm^	0.14 ± 0.02^p^	0.02 ± 0.01°^p^
C6	0.53 ± 0.05^fg^	6.92 ± 0.05^a^	1.02 ± 0.09^k^	4.45 ± 0.06^b^	0.40 ± 0.07^n^	0.43 ± 0.05°	0.00 ± 0.00^p^
E1	0.65 ± 0.03^de^	0.29 ± 0.05 ^m^	1.59 ± 0.06^i^	1.03 ± 0.03^n^	1.99 ± 0.06^ef^	5.81 ± 0.07^e^	3.12 ± 0.07 ^g^
E2	0.49 ± 0.05^gh^	0.38 ± 0.02 ^lm^	2.18 ± 0.10^cde^	0.97 ± 0.03^no^	2.80 ± 0.10^a^	7.15 ± 0.11^a^	5.26 ± 0.06^b^
E3	0.41 ± 0.03^hi^	0.14 ± 0.05^n^	1.16 ± 0.03^j^	0.69 ± 0.06^p^	2.20 ± 0.09 ^cd^	6.60 ± 0.08^c^	4.61 ± 0.11^d^
E4	1.01 ± 0.11^b^	0.35 ± 0.02 ^lm^	1.93 ± 0.04^gh^	0.87 ± 0.05^o^	2.17 ± 0.11 ^cd^	6.33 ± 0.05^d^	4.33 ± 0.10^e^
E5	0.41 ± 0.04^hi^	0.44 ± 0.10 ^l^	1.60 ± 0.06^i^	0.50 ± 0.05^q^	2.59 ± 0.10^b^	4.19 ± 0.04 ^h^	7.30 ± 0.08^a^
E6	0.40 ± 0.02^hi^	0.37 ± 0.02 ^lm^	2.25 ± 0.06^c^	1.25 ± 0.06 ^m^	2.79 ± 0.09^a^	6.84 ± 0.06^b^	4.30 ± 0.06^e^
E7	0.62 ± 0.06^ef^	0.86 ± 0.03^j^	3.85 ± 0.13^a^	3.34 ± 0.06 ^g^	2.28 ± 0.03^c^	5.61 ± 0.09 ^f^	2.69 ± 0.01 ^h^
E8	0.30 ± 0.02^ij^	0.93 ± 0.05^j^	2.91 ± 0.02^b^	2.51 ± 0.03^i^	1.76 ± 0.13^gh^	1.60 ± 0.06 ^l^	1.31 ± 0.08^j^
E9	0.31 ± 0.02^ij^	0.31 ± 0.06 ^m^	1.67 ± 0.11^i^	1.95 ± 0.04^k^	2.19 ± 0.10 ^cd^	5.02 ± 0.10 ^g^	5.10 ± 0.09^c^
E10	1.00 ± 0.12^b^	0.56 ± 0.02^k^	1.64 ± 0.03^i^	1.52 ± 0.05 ^l^	1.64 ± 0.03^hi^	5.13 ± 0.08 ^g^	1.80 ± 0.03^i^
E11	0.62 ± 0.06^ef^	1.13 ± 0.04^i^	2.06 ± 0.10^ef^	2.34 ± 0.08^j^	1.98 ± 0.03^ef^	3.78 ± 0.10^i^	3.19 ± 0.11 ^g^
E12	0.75 ± 0.05 ^cd^	0.89 ± 0.06^j^	1.90 ± 0.10^gh^	1.93 ± 0.04^k^	1.96 ± 0.06 ^f^	4.30 ± 0.01 ^h^	3.39 ± 0.08 ^f^
SW1	0.55 ± 0.04^efg^	3.55 ± 0.05^d^	1.60 ± 0.04^i^	4.06 ± 0.05 ^cd^	1.48 ± 0.05^j^	0.89 ± 0.04^n^	0.18 ± 0.03^n^
SW2	0.79 ± 0.12^c^	4.71 ± 0.04^c^	1.83 ± 0.08 ^h^	5.55 ± 0.07^a^	1.17 ± 0.01^k^	0.39 ± 0.08^o^	0.09 ± 0.02^nop^
SW3	0.41 ± 0.04^hi^	5.17 ± 0.10^b^	1.00 ± 0.03^k^	3.98 ± 0.08^d^	1.80 ± 0.04 ^g^	3.27 ± 0.03^j^	0.88 ± 0.10 ^l^
SW4	0.82 ± 0.05^c^	2.61 ± 0.05^e^	2.08 ± 0.10^def^	3.47 ± 0.08 ^f^	1.54 ± 0.13^ij^	2.15 ± 0.06^k^	0.65 ± 0.08 ^m^

Note: Data were presented as mean ± standard deviation. The same lower-case letters within each column indicate a non-significant difference (*p* > 0.05); different lower-case letters within each column indicate a significant difference (*p* < 0.05).

The concentrations of various bufogenins were related to each other (Table 2). Generally, with increasing CBG concentration, the concentrations of both BL and RBG significantly increased, but the TBG and CFL concentrations decreased.

**Table 2:** The correlation of seven bufogenins concentrations.

Bufogenins		GB	TBG	BFL	CFL	BL	CBG	RBG
GB	r	1						
	*p*	—						
TBG	r	0.002	1					
	*p*	0.992	—					
BFL	r	−0.143	−0.022	1				
	*p*	0.525	0.922	—				
CFL	r	−0.119	0.870**	0.029	1			
	*p*	0.597	0.000	0.899	—			
BL	r	0.135	−0.757**	0.091	−0.767**	1		
	*p*	0.549	0.000	0.687	0.000	—		
CBG	r	0.316	−0.818**	−0.020	−0.797**	0.891**	1	
	*p*	0.152	0.000	0.930	0.000	0.000	—	
RBG	r	0.073	−0.871**	−0.034	−0.883**	0.910**	0.845**	1
	*p*	0.747	0.000	0.879	0.000	0.000	0.000	—

Note: *p < 0.05; **p < 0.01.

### Geospatial distribution characteristics of the concentrations and proportions of bufogenins

The variation in bufogenin concentrations was significantly related to geographic longitude (*p < *0.01) but not to latitude (*p* > 0.05). Specifically, the concentrations of TBG and CFL were negatively correlated with longitude (r = −0.787, *p* < 0.01 and r = −0.836, *p* < 0.01, respectively), while conversely, the concentrations of BL, CBG and RBG were positively correlated with longitude (r = 0.708, *p* < 0.01; r = 0.685, *p* < 0.01 and r = 0.851, *p < *0.01, respectively). The total bufogenin concentration was positively correlated with longitude (r = 0.560, *p* < 0.01).

**Figure 3:**
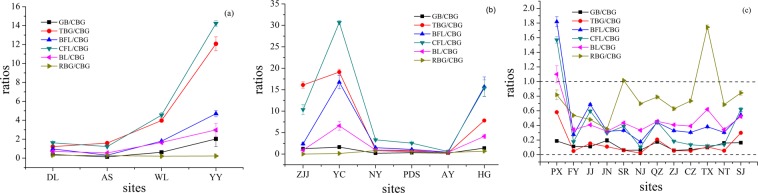
The proportion of bufogenins in Asian toads. (**a**) southwestern China sites; (**b**) central China sites; (**c**) eastern China sites. The sites were sorted by longitude from low to high.

The composition and proportions of defensive chemicals can markedly affect deterrent effects^20^. This study investigated the geospatial distribution characteristics of the GB/CBG, TBG/CBG, BFL/CBG, CFL/CBG, BL/CBG and RBG/CBG ratios, as shown in Fig. 3. With increasing longitude, the line chart of all the ratio values except the RBG/CBG presented an increasing trend at the southwestern China sites, and the line chart of all the ratio values displayed cross-fluctuation trends at the central and eastern China sites. However, except for RBG/CBG, the fluctuation trends of all ratios were consistent at the central China sites, and only the fluctuation trends of BFL/CBG and BL/CBG were identical at the eastern China sites. Moreover, TBG/CBG and CFL/CBG were always significantly higher than GB/CBG, BL/CBG and RBG/CBG at the southwestern and central China sites. In addition, at the twelve eastern China sites, except for PX, SR and TX, all six ratio values from different sites were less than 1, which indicated that CBG was the richest component among the bufogenins in the eastern regions.

### Geographic clustering of toad toxins based on chemometrics

**Figure 4:**
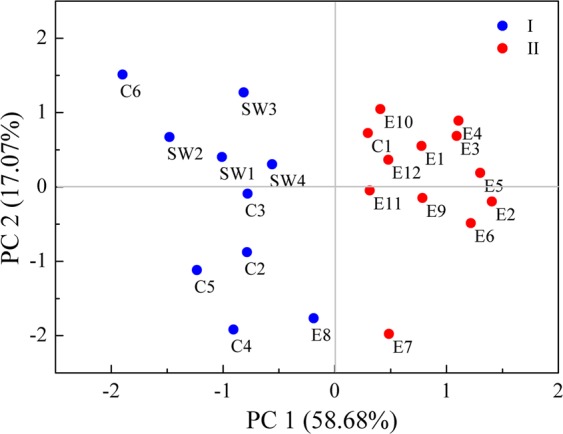
The PCA score plot of the toad toxin samples collected from twenty-two geographical origins. Each point represented an individual sample, and the points of the same color (blue or red) were similar in terms of bufogenins composition.

The principal component analysis (PCA) score plot of the toad toxin samples (Fig. 4) showed a clear separation among the samples from different geographical origins, with the first two components explaining 75.75% of the total variance. Based on their chemical profiles, the toad toxin samples were classified into two main clusters (I and II) encompassing the twenty-two geographical origins: 1) C2-C6, SW1-SW4 and E8 were cluster I, and 2) C1, E1-E7 and E9-E12 were cluster II. The toxin samples C1-C6 and SW1-SW4 were obtained from central and southwestern China, and samples E1-E12 were obtained from eastern China. From a geographical perspective, C1 was obtained from Anyang, which is located in north of the Huaihe River, in the northernmost part of Henan Province; E8 was collected from Pingxiang, located in the western part of Jiangxi near Hunan Province. The concentrations of bufogenins from these two locations (Anyang and Pingxiang) were similar to those in the adjacent regions but different from those in other regions of the provinces.

**Figure 5:**
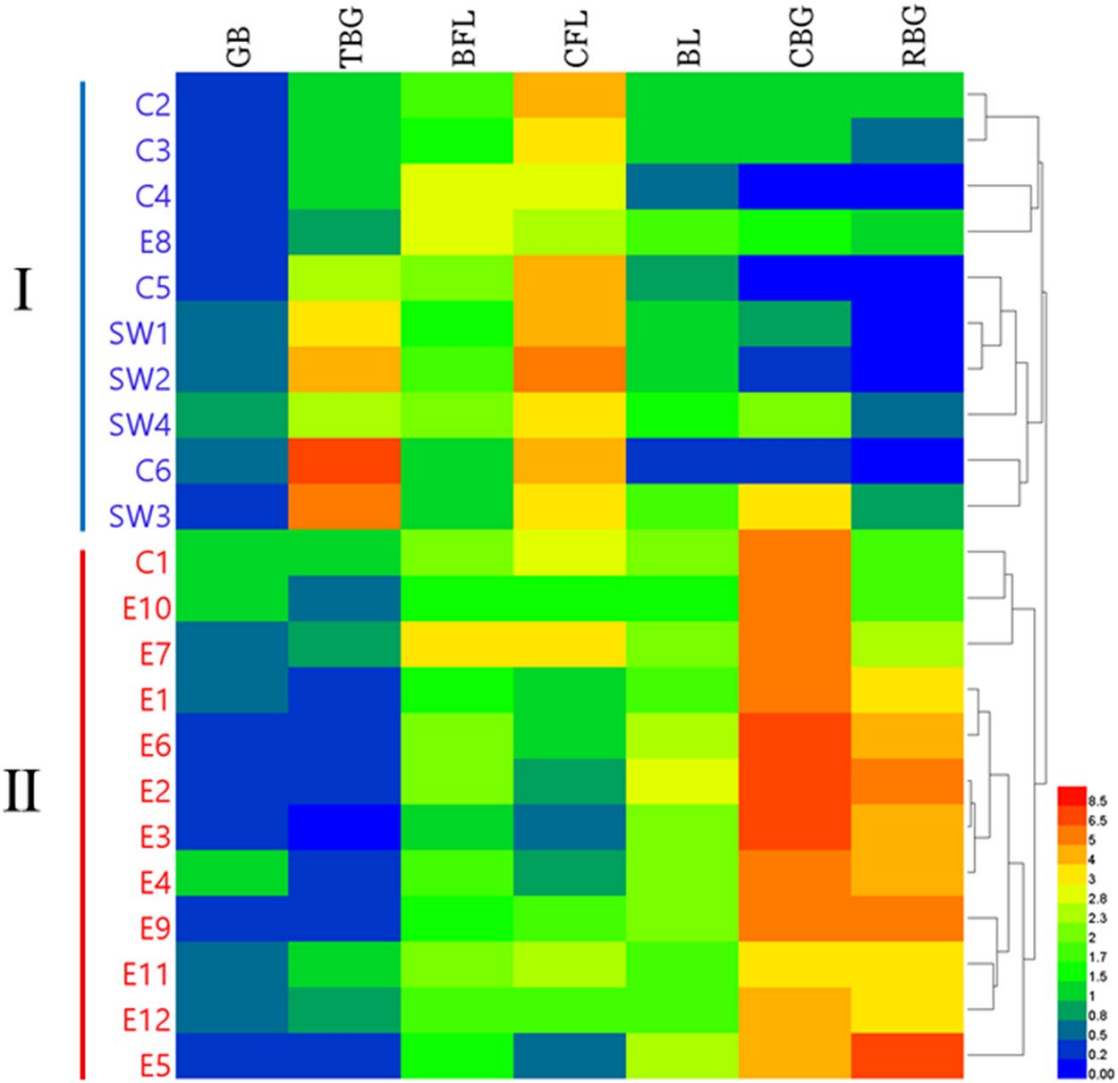
The hierarchical clustering heatmap (HCH) of the toad toxin samples collected from twenty-two geographical origins. The colors indicated the gradually up-regulated level of bufogenins concentrations from blue (minimum) to red (maximum).

The classification results based on the hierarchical clustering heatmap (HCH) were consistent with the PCA results (Fig. 5). The distributions of the seven bufogenins in twenty-two geographical origins was clearly displayed by the heatmap. The distribution characteristics of bufogenins in different clusters were distinctly different. The concentrations of TBG and CFL were significantly higher in cluster I than in cluster II, whereas the concentrations of CBG and RBG were higher in cluster II than in cluster I. In six out of ten samples in cluster I, CFL constituted the largest proportion, ranging between 26.04% and 40.41%. However, CBG was the predominant active constituent in cluster II except in E5 and E9, ranging from 25.05% to 41.75%.

**Figure 6:**
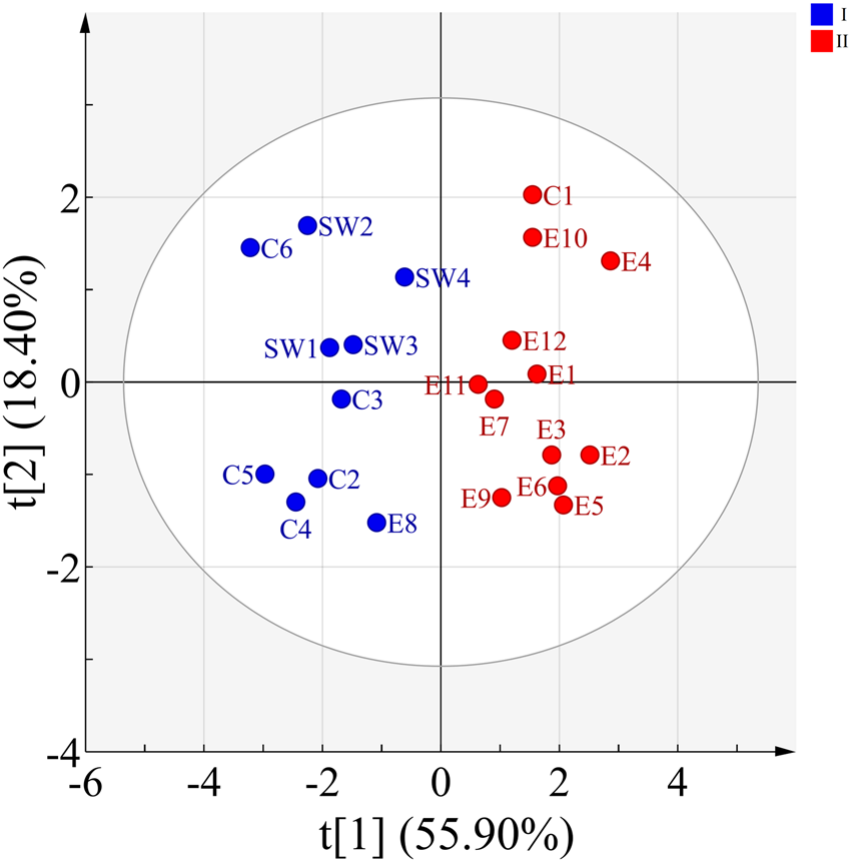
The OPLS-DA score plot of the toad toxin samples collected from twenty-two geographical origins. Each point represented an individual sample, and the points of the same color (blue or red) were similar in terms of bufogenins composition.

The results of orthogonal partial least squares discriminant analysis (OPLS-DA) were consistent with those of PCA and HCH. The OPLS-DA score plot (see Fig. 6) could also be divided into two zones according to the twenty-two sites with R2Y = 0.871 and Q2 = 0.831, demonstrating the good classification and prediction ability of the model.

### The effects of climatic factors on bufogenins

All 8 climatic variables had significant effects on bufogenin concentration (*r*_*i*_> 0.5; Table 3). Among them, PRE_Time and PRE_Max showed the most significant effect on bufogenin accumulation, followed by RHU_Avg. The results from January 2018 (*r*_*i*_-Jan.) were essentially in agreement with those from February 2018 (*r*_*i*_-Feb.).

**Table 3:** The grey relational grades *r*_*i*_ between climatic factors and the total bufogenin concentration.

Climatic factors	Jan.	Rank	Feb.	Rank
TEM_Avg	0.6192	7	0.6001	8
TEM_Max	0.6257	4	0.6442	5
TEM_Min	0.6230	5	0.6332	6
RHU_Avg	0.6596	3	0.7049	3
PRE_Time	0.7253	2	0.7110	1
PRE_Max	0.7356	1	0.7090	2
GST_Max	0.6132	8	0.6161	7
GST_Min	0.6217	6	0.6859	4

**Figure 7:**
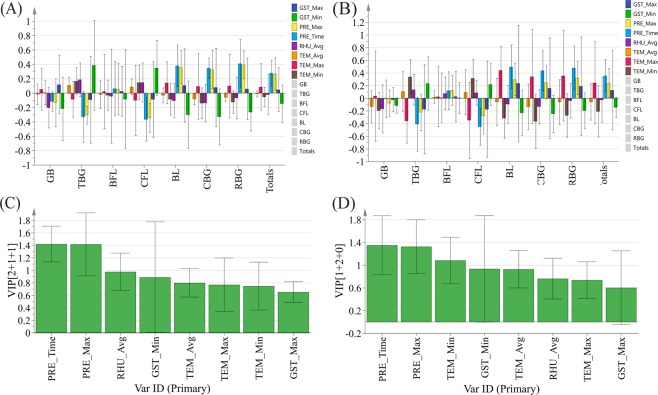
The standardized regression coefficient plots (**A**: Jan.; **B**: Feb.) and VIP plots (**C**: Jan.; **B**: Feb.) of OPLS.

Based on the coefficient plots of the OPLS model (Fig. 7A,B), the extent and direction of the influence of the eight climatic factors on the seven bufogenins were significantly different. The concentrations of GB and BFL were minimally affected by the eight climatic factors (|r| < 0.2). In the other five bufogenins, because of the correlation between the components, the directions of the influence of the climatic factors on TBG and CFL were the same. The directions of influence on BL, CBG and RBG were also the same, but the intensity of the influence was not completely consistent in our study. Moreover, these two groups of toxic ingredients were affected by climatic factors in opposite directions. Specifically, PRE_Time and PRE_Max were both negatively associated with TBG and CFL but positively correlated with BL, CBG and RBG, and the effect on total bufogenin concentration was consistent with the effects on the latter three components. That is, the total bufogenin concentration increased with increasing precipitation. The main climatic factors influencing the concentration were PRE_Time and PRE_Max, which had variable importance in the projection (VIP) values greater than 1 (Fig. 7C,D). These results were consistent with those of the GRA.

## Discussion

We found that the concentrations and proportions of the main defensive chemicals in adult Asian toads from different geographical regions were highly variable. Compared to toads from central and southwestern China, the toads from eastern China had higher concentrations of CBG and RBG but lower concentrations of TBG and CFL. This variation in bufogenin concentrations showed a specific spatial structure related to the origins of the bufogenins, and the degree of variation increased significantly with increasing geographical distance, suggesting that toads with different bufogenin profiles are nonrandomly distributed among geographical regions.

Interestingly, despite the geographical variation in chemical defence, the concentrations of the various compounds in the toxin blend were related to each other. Therefore, we hypothesized that positive or negative correlation of various toxin components result from constitutive defences in toads, whereas inducible plasticity determines the dominant compounds of the defensive toxin blend. In terms of why the dominant compounds in the toxin blend from different habitats were significantly different, we argue that environmental factors in different regions may regulate the synthesis of these compounds. Many studies have demonstrated that the biosynthesis of the bufogenin component starts with common precursor molecules^20,31,32^, and the negative correlation between the production levels of certain toxins may be the result of competition for these limited precursors. Additionally, the synthesis of these compounds may involve different biosynthesis pathways, which may be selectively accessed in different environmental conditions. Some studies have indicated that bufogenins may also be transformed by certain microorganisms^33,34^, and the correlations between different chemical components may result from biotransformation of the toad skin microbiome in different habits.

In addition to elucidating the variation in chemical defences, the relationships between defensive chemicals and environmental factors are also relevant for understanding the consequences of this variation. As the mixed defensive toxins were obtained from different adult toads at each geographical site, this regular pattern of chemical defence phenotypic plasticity induced by geographical factors was not related to the gender, age, or size of the individual toads. Although predators and competitors in different regions may have certain effects on the variation in defensive toxin production^8,12,34^, the density and type of these species in different regions vary greatly^7,34,35^. Accordingly, we conclude that this regular variation in geographical environment was not mainly induced by the presence of these species.

The variation may be primarily attributable to the climatic factors in different geographic locations. We found that the relationships between climate variables and bufogenin concentration, despite various climatic drivers, exert different influences on toxin production. Our results also showed that the total bufogenin concentration was positively correlated with precipitation, demonstrating that toxin production exhibits climate-induced plasticity.

Although the biosynthesis pathway of these defensive compounds is unknown^32^, previous studies found that the amount of biosynthesis-related energy absorbed by toads mainly depends on weather conditions as modified by habitat structure^36^. The habitats chosen by toads will determine their body temperature and hydration state, which can have direct physiological, behavioural and functional consequences^37–39^. As an amphibian, toads rely particularly on humid environments. In addition, toads obtain water primarily by osmotic absorption across their integument under a favourable osmotic gradient because moist skin is highly permeable^40^. The highly vascularized abdominal skin contains a seat patch or pelvic patch^41,42^, which is specialized for rapid water absorption to restore plasma volume and osmolality^40^. Therefore, the precipitation and humidity of the external environment will directly affect the water balance in toads, which in turn will have a certain impact on the metabolism. Previous studies found that toad tadpoles tended to contain higher concentrations of defensive toxin in ponds that were less likely to dry out^7^. As an alternative explanation for the positive correlation between total bufogenin concentration and precipitation, it is possible that toads retain the trait of tadpoles by producing more bufogenins in a humid environment.

Climate-induced chemical defence plasticity may have major ecological consequences. First, the defensive chemicals produced by animals can provide protection against natural enemies^11,15^, and the composition of the defensive toxin mixture may substantially affect its function or deterrence efficiency^20^. Such climate-induced plasticity may reflect a diversity of chemical defence strategies, thereby possibly enhancing anti-predator efficacy. For example, individuals with certain compositions of toxic chemicals may defend against enemies with corresponding toxin susceptibilities^28^. However, the survival probability of animals may decrease if toxin production is reduced by unfavourable climate conditions at a particular time. Based on our current results, extreme drought may lead to a reduction in the main toxin concentration, and prey species exposed to the environment may increase their predation risk. Recent studies have suggested that most species are facing a high risk of extinction^18,43,44^, and climate change is an important factor affecting the survival of various species^45–47^. Relatively little is known about the demographic mechanisms through which climatic variation affects population dynamics in species^48^. Our results show that climate might indirectly and partially reflect the species survival probability by regulating chemical defences. In addition, climate-induced the plasticity of chemical defence may impact the fitness of species. A previous experimental study showed that the synthesis of defensive chemicals imposes a substantial cost related to energetic trade-offs between compound production and reproduction^31^. Animals that invest more in toxin production do so at the expense of reproductive investment^31^. Further testing is needed to investigate whether climate-induced high levels of toxin production will reduce the reproductive success of both males and females.

In conclusion, our results are the first to document that toads phenotypically adjust their chemical defences in response to variable climate and geographical conditions. The observation that toads produced a high concentration of the defensive toxin in regions with considerable precipitation indicated that bufogenin production may be related to water in the environment. These results, combined with those of previous studies^5,7,10^, suggest the existence of multiple approaches to inducible chemical defences. Studies on the environmental factors regulating toxin synthesis and the costs and mechanisms of toxin production, clarifying the deterrent efficacy of toxin mixtures constituted by diverse compositions in facing different environmental risks will be favoured for further understanding of the evolution and ecology of chemical defences.

## Materials and Methods

### Study species and sites

The study species was Asian toads (*Bufo gargarizans*), the most common toad in China. Adult toads were collected as samples in early March 2018 from a total of twenty-two sites in China. Study sites were classified into three groups according to geographic location: central, eastern and southwestern China. These regions are located between latitudes of 25.61°−36.65° and longitudes of 100.27°−121.23° (Table 4). We collected the animals in accordance with the permits issued by the China’s State Forestry Administration (Department of Wildlife Conservation) and the Jiangsu Forestry Bureau (Su [Huai Yu] Forest Wild permission number 2018002). The study was further approved by the Ethics Review Committee of Experimental Animal Welfare, Zhejiang University. All methods were carried out in accordance with relevant guidelines and regulations.

**Table 4:** The samples collection information.

Samples No.	Habitats (City/Province)	Origin abbreviation	Regions	Longitude	Latitude
C1	Anyang/Henan	AY	Central	114.39	36.09
C2	Nanyang/Henan	NY	Central	112.53	32.99
C3	Pingdingshan/Henan	PDS	Central	113.19	33.77
C4	Huanggang/Hubei	HG	Central	114.87	30.45
C5	Yichang/Hubei	YC	Central	111.29	30.69
C6	Zhangjiajie/Hunan	ZJJ	Central	110.48	29.12
E1	Fuyang/Anhui	FY	Eastern	115.81	32.89
E2	Changzhou/Jiangsu	CZ	Eastern	119.97	31.81
E3	Nanjing/Jiangsu	NJ	Eastern	118.80	32.06
E4	Nantong/Jiangsu	NT	Eastern	120.89	31.98
E5	Taixing/Jiangsu	TX	Eastern	120.25	32.24
E6	Zhenjiang/Jiangsu	ZJ	Eastern	119.43	32.19
E7	Jiujiang/Jiangxi	JJ	Eastern	116.00	29.71
E8	Pingxiang/Jiangxi	PX	Eastern	113.85	27.62
E9	Shangrao/Jiangxi	SR	Eastern	117.94	28.45
E10	Jinan/Shandong	JN	Eastern	117.12	36.65
E11	Songjiang/Shanghai	SJ	Eastern	121.23	31.03
E12	Quzhou/Zhejiang	QZ	Eastern	118.86	28.97
SW1	Wulong/Chongqing	WL	Southwestern	107.76	29.33
SW2	Youyang/Chongqing	YY	Southwestern	108.77	28.84
SW3	Anshun/Guizhou	AS	Southwestern	105.95	26.25
SW4	Dali/Yunnan	DL	Southwestern	100.27	25.61

The parotid glands of the toads were scraped moderately with an aluminium scraper. The milk-like fresh secretions obtained were placed into clean glass dishes. After this procedure, the wounds of the animals were gently wiped with erythromycin ointment to reduce inflammation. Then, the animals were returned to the land. The animals should not be placed directly in the water in order to prevent the wounds infection and inflammation. All collected toxin samples were placed in coolers and immediately transported to the laboratory. The samples were placed in the freezer compartment of a refrigerator at −20 °C and used within two days to avoid compositional shifts during storage. The voucher specimens were deposited in the laboratory of the Institute of Modern Chinese Medicine, Zhejiang University.

### Reagents and standards

HPLC grade of methanol and acetonitrile were supplied by J&K Scientific Ltd. (Beijing, China), HPLC grade of formic acid were obtained from Aladdin Bio-Chem Technology Co., Ltd. (Shanghai, China), Ultrapure water was generated by Milli-Q water purification system (Hangzhou, China). The reference standards (Fig. 1) of gamabufotalin (GB), telocinobufagin (TBG), bufotalin (BFL), cinobufotalin (CFL), bufalin (BL), cinobufagin (CBG) and resibufogenin (RBG) were purchased from Shanghai yuanye Bio-Technology Co., Ltd. (Hanghai, China).

### Preparation of standard solutions

A stock solution of seven standards was prepared by dissolving accurately weighed standards in methanol. Working standard solutions for calibration curves were obtained by diluting the mixed standard solution with methanol to obtain a series of different concentrations of these analytes. All solutions were stored in a refrigerator at 4 °C.

### Preparation of sample solutions

0.2 g fresh secretions sample obtained from the parotoid glands of toad was accurately weighted and extracted with 20 mL methanol through heating reflux for one hour. The extraction solutions were cooled at room temperature and then the lost solvent were replenished with methanol. All of the samples solutions were centrifuged at 4,000 rpm for 5 min and then the supernatant was filtered through a 0.45 μm filter membrane before HPLC analysis.

### Chromatographic analysis

Methanol extracts of samples were prepared as described above. The bufogenins concentration were determined by the method established previously^26^. The chromatographic analysis was primarily performed by an Agilent 1200 system (Agilent Technologies, Tokyo). Analytes were separated on a XBridge^®^ Shield RP C_18_ column (4.6 × 250 mm, 5 µm). The water (A) containing 0.1% formic acid (v/v) and acetonitrile (B) were used as mobile phase. The gradient elution program was: 0–5 min, 3–5% B; 5–10 min, maintain 5% B; 10–14 min, 5–35% B; 14–22 min, stay 35% B; 22–32 min, 35–40% B; 32–37 min, 40–46% B; 37–47 min, 46–50% B; 47–50 min, 50–3% B. The total run time was 50 min. The elution was performed with a flow rate of 0.7 ml/min. The column temperature was maintained at 30 °C. The detection wavelength was set at 296 nm and the injection volume was 10 μL. The seven compounds concentration were expressed with relative % (mg/100 mg).

### Climate data

The eight main climatic factors were average temperature (°C) (TEM_Avg), maximum temperature (°C) (TEM_Max), minimum temperature (°C) (TEM_Min), average relative humidity (%) (RHU_Avg), 20–20 time precipitation (mm) (PRE_Time), maximum continuous precipitation (mm) (PRE_Max), maximum ground temperature (°C) (GST_Max) and minimum ground temperature (°C) (GST_Min). Data related to the climatic factors were obtained from the China Meteorological Administration. Given that the biosynthesis of defensive chemicals takes time^31^, we selected daily climate data for the first two months before the toxin samples collection (January and February 2018) and calculated the monthly average values of the eight climatic factors at the twenty-two geographical sites (Table S1).

### Data analysis

Climate system can usually be considered as grey system due to their high complexity and unpredictable change. It’s considerably difficult to explore the combined influence of multiple climatic factors on toxic components. In view of this, this paper utilized grey relational analysis (GRA) and multivariate analysis (MVA). GRA, as a powerful tool dealing with problems of small samples and imprecise information, has been widely applied in many fields^49,50^. But the correlation coefficient in the GRA can only indicate the influence degree of the compared series to the reference series, and cannot reflect the influence direction (positive or negative)^51^. Therefore, the influence direction of various climatic factors on bufogenin accumulation were further explored by MVA. MVA can re-establish a new set of features by extracting highly correlated projections of data representations from input and output spaces. MVA includes PCA, OPLS-DA, and OPLS^52–54^. PCA is an unsupervised method and is very favourable for preliminary classification^54^. OPLS is a popular supervised method and is being increasingly adopted as an alternative to partial least squares regression (PLS) due to better generalization^55^. The multiple climatic factors and variety of bufogenin concentrations, as high-dimensional variables, can be analysed by these methods.

### Grey relational analysis

To evaluate the importance of various climatic factors on the concentration of bufogenins, independent input variables, i.e.TEM_Avg, TEM_Max, TEM_Min, RHU_Avg, PRE_Time, PRE_Max, GST_Max and GST_Min, were chosen as the compared series and defined as:

1 $${X}_{i}=\{{X}_{i}(k)|i=1,2,\ldots ,m;k=1,2,\ldots ,n\},$$

The output variable (the total bufogenins concentration) was set as the reference series and expressed as:

2 $${X}_{0}=\{{X}_{0}(k)|k=1,2,\ldots ,n\},$$

where *m* is the total number of indexes to be considered, and *n* is the total number of samples (*m* = 8, *n* = 22 in this experiment).

Due to the different dimensions of the original data, it is necessary to standardize the original data. In this experiment, raw data of multiple climatic factors and the total concentration of seven bufogenins were normalized by SPSS 20.0 software for Z-Score standardization to obtain a series of dimensionless normalized data.

The grey relational coefficients reflects the relationship between climatic factors and the total bufogenins concentration. The calculation formula of grey relational coefficients is:

3 $${\xi }_{i}(k)=\frac{{\min }_{i}{\min }_{k}|{X}_{0}^{\ast }(k)-{X}_{i}^{\ast }(k)|+\rho \,{\max }_{i}{\max }_{k}|{X}_{0}^{\ast }(k)-{X}_{i}^{\ast }(k)|}{|{X}_{0}^{\ast }(k)-{X}_{i}^{\ast }(k)|+\rho \,ma{x}_{i}\,ma{x}_{k}|{X}_{0}^{\ast }(k)-{X}_{i}^{\ast }(k)|},$$

${X}_{0}^{\ast }(k)$ and ${X}_{i}^{\ast }(k)$ are the standardization value of *X*_0_ (*k*) and *X*_*i*_ (*k*), respectively. *ρ* (0 < *ρ* ≤ 1) is a distinguishing coefficient to adjust the range of the comparison environment, normally used value is *ρ* = 0.5.

Finally, the value of the grey correlation degree were obtained by calculating the mean values of all the grey relational coefficients.

4 $${r}_{i}=\frac{1}{n}{\sum }_{k=1}^{n}{\xi }_{i}(k),$$

Above steps were carried out by Matlab R2018a.

### Statistical analyses

All experiments were performed in triplicate, and the values are expressed as the mean ± standard deviation. The raw data on multiple climatic factors and the bufogenin concentrations were normalized by Z-score standardization to obtain a series of dimensionless normalized data. The obtained data were analysed by one-way ANOVA of Duncan’s multiple comparison test and Spearman’s two-tailed correlation analysis using SPSS 20.0 software. The level of significance for all hypothesis tests (*p*) was 0.05. The bufogenin concentrations were used as the variables, and the toxin samples were the observation IDs. The obtained data were imported into SPSS 20.0 software for PCA to reveal the intrinsic relations in the data set and to diagnose any possible outlier. In the HCH analysis, the data were processed based on the average linkages using HemI (Heatmap Illustrator, version 1.0). Then, OPLS-DA was performed to evaluate the robustness and accuracy of the classification results using SIMCA-P version 14.0. Finally, the relationships between climatic factors and bufogenin concentration were evaluated by the regression coefficient and VIP of the OPLS. The regression coefficient can reflect the positive or negative effect of the X variables (eight climatic factors) on the Y variables (the concentrations of single bufogenins and the total bufogenin concentration). In general, VIP values of the X variables that are greater than 1 are considered decisive indicators with significant influence on the Y variables^56^.

## Supplementary information

Supplementary Dataset 1
